# The Role of Next-Generation Sequencing in the Management of Patients with Suspected Non-Ischemic Cardiomyopathy after Syncope or Termination of Sudden Arrhythmic Death

**DOI:** 10.3390/genes15010072

**Published:** 2024-01-05

**Authors:** Damijan Vokač, Špela Stangler Herodež, Danijela Krgović, Nadja Kokalj Vokač

**Affiliations:** 1 Department of Cardiology and Angiology, Division of Internal Medicine, University Medical Centre Maribor, 2000 Maribor, Slovenia; damijan.vokac@ukc-mb.si; 2 Clinical Institute for Genetic Diagnostics, University Medical Centre Maribor, 2000 Maribor, Slovenia; spela.stanglerherodez@ukc-mb.si (Š.S.H.); danijela.krgovic@ukc-mb.si (D.K.); 3 Medical Faculty, University of Maribor, 2000 Maribor, Slovenia

**Keywords:** malignant cardiac arrhythmias, sudden death, non-ischemic cardiomyopathy, next-generation sequencing

## Abstract

Cardiac arrhythmias and sudden death are frequent in patients with non-ischemic cardiomyopathy and can precede heart failure or additional symptoms where malignant cardiac arrhythmias are mostly the consequence of advanced cardiomyopathy and heart failure. Finding these subgroups and making an early diagnosis could be lifesaving. In our retrospective study, we are presenting arrhythmic types of frequent cardiomyopathies where an arrhythmogenic substrate is less well defined, as in ischemic or structural heart disease. In the period of 2 years, next-generation sequencing (NGS) tests along with standard clinical tests were performed in 208 patients (67 women and 141 men; mean age, 51.2 ± 19.4 years) without ischemic or an overt structural heart disease after syncope or aborted sudden cardiac death. Genetic variants were detected in 34.4% of the study population, with a significant proportion of pathogenic variants (P) (14.4%) and variants of unknown significance (VUS) (20%). Regardless of genotype, all patients were stratified according to clinical guidelines for aggressive treatment of sudden cardiac death with an implantable cardioverter defibrillator (ICD). The P variant identified by NGS serves for an accurate diagnosis and, thus, better prevention and specific treatment of patients and their relatives. Results in our study suggest that targeted sequencing of genes associated with cardiovascular disease is an important addendum for final diagnosis, allowing the identification of a molecular genetic cause in a vast proportion of patients for a definitive diagnosis and a more specific way of treatment. VUS in this target population poses a high risk and should be considered possibly pathogenic in reanalysis.

## 1. Introduction

Genomic analyses have brought great progress in clinical medicine [1]. According to the 2023 ESC Management Guidelines, genetic testing and genetic counseling are performed for the affected individual with cardiomyopathy based on the phenotypic definition of the disease, with the caveat that the finding is clinically useful only if a genetic variant of pathogenic/likely pathogenic (P/LP) is found [2]. For an increasing number of conditions, a genetic diagnosis can provide prognostic information, and a genetic diagnosis can directly stratify the choice of therapy. However, there is still a gap between understanding the complexity of NGS analysis data and its use in diagnostics and treatment [3]. Clinical phenotyping is crucial in the interpretation of genetic results after NGS analyses. There are different approaches for the genetic testing of causative candidate genes for the development of cardiovascular diseases, from target sequencing of disease-associated genes to more complex whole exome sequencing analyses or even whole genome sequencing analyses [4].

Cardiac arrhythmias are frequent in patients with non-ischemic cardiomyopathy (CMP) and channelopathy (CHP) and can precipitate sudden cardiac death far before heart failure or additional symptoms set up. In our clinical patient group, the most frequent non-ischemic cardiomyopathy is dilated cardiomyopathy (DCM). After the ClinGen curation scoring framework, there were 19 genes with substantial evidence supporting a role in monogenic DCM, 11 genes with strong-evidence classifications, and 7 genes with moderate-evidence classifications [5,6]. In the majority of DCM cases, malignant cardiac arrhythmias, mostly ventricular tachyarrhythmias (VT), are a nonspecific consequence of advanced cardiomyopathy and heart failure. Although in many cases, they could present a primary onset of hereditary disease and precede symptoms [7,8]. In these subgroups of patients, early diagnosis before or just after the primary episode of ventricular tachyarrhythmia or the survival of sudden death due to tachyarrhythmia could be lifesaving. The arrhythmogenic entity of ischemic or post-infarctional cardiomyopathy is in clinical practice well-defined regarding the need for implantation of ICD or radiofrequency (RF) ablation of arrhythmogenic substrate procedure [9], but it is less well-defined for the need and prophylactic use in patients with cardiomyopathies, especially those without advanced symptoms, modest heart failure, and preserved left ventricular ejection fraction (LVEF) > 40% [10]. Ventricular arrhythmias could be a non-specific consequence of an overt DCM or HCM in association with secondary structural and electrical heart chamber remodeling, but they could also be primary manifestations of the disease process itself [11,12]. In present clinical practice, clinical stratification criteria are mostly dependent on the exclusion of structural heart diseases and arrhythmogenic substrates. For exclusion purposes, we use several clinical cardiac procedures, including clinical history, according to present guidelines [2], noninvasive procedures such as standard 12-lead electrocardiography (ECG) with emphasis on specific characteristics such as QRS fragmentation and T-wave changes, Holter monitoring, left ventricular measurement and left ventricular ejection fraction (LVEF) by echocardiography, autonomic nervous function activity testing, magnetic resonance imaging (MRI), and invasive procedures such as coronary angiography (CA) and electrophysiological testing (EPS) [11,12,13]. Clinical changes are often overlooked in patients with no or very modest symptoms, and they could later develop an overt clinical disease and a relapse of malignant ventricular arrhythmias. On the other hand, several symptomatic patients with suspected presyncope could have normal test outcomes during the non-invasive or invasive diagnostic cardiac procedures, giving a rather false impression of normal heart physiology. In this sense, NGS testing could be very helpful in detecting the causing genetic variants [9,10,13]. Recognition of arrhythmogenic genotypes among these groups could be clinically feasible and important because early recognition and aggressive intervention could be lifesaving for the patient [14]. 

In our study, a gene panel of already known candidate genes was used, which enables a comprehensive, cost-effective solution for identifying causal variants implicated in inherited cardiac conditions. In the presented article, we provide some examples of important clinical assessments for the correct interpretation of genetic variants [15,16].

## 2. Materials and Methods

During a period of 2 years, NGS testing was performed in 208 patients (mean age, 51.2 ± 19.4 years, 67/141 (F/M)) without ischemic or an overt structural heart disease after syncope, after spontaneously terminated or converted ventricular tachycardia (VT) in sinus rhythm, or after survival of sudden cardiac death due to ventricular fibrillation (VF). Patients were risk-stratified according to standard clinical criteria [12], and only patients with normal echocardiography, normal heart MRI, and normal CA were included in the study [14]. All the patients with overt structural heart disease were excluded. In 92 patients after aborted sudden death due to VF or positive inducible electrophysiologic testing and high probability for relapse of VT/VF, an ICD implantation was immediately performed under clinical guidelines [9,10]. In 21 patients with syncope and after an inducible electrophysiologic test with stable sustained monomorphic VT, an RF ablation procedure was performed under strict clinical criteria, and these patients were carefully followed. Other patients remained on optimal medical therapy and were carefully followed by non-invasive testing, such as Holter monitoring or endless loop recorder implantation [12].

Clinical testing includes a history of the disease among the patients and family history, with an emphasis on arrhythmogenic and heart failure symptoms amongst family members, information on familiar forms of cardiomyopathies and channelopathies, conduction and structural abnormalities, atrial and ventricular arrhythmias, and especially a history of sudden death. Previously recorded ECGs of the patient and relatives were studied. The clinical examination included a 12-lead ECG, echocardiography, and 24-h ECG, as well as cardiac magnetic resonance imaging for further analysis of ventricular size and function and assessment of the possible presence of ventricular fibrosis. In all the patients, ischemic heart disease was excluded by CA. Further analysis included standard EPS and programmed stimulation upon clinical indication and careful biochemical biomarker tests, including heart troponin leakage. Patients were followed up for at least 5 years, and during this period, the described clinical tests were repeated [12].

### Next-Generation Sequencing (NGS)

NGS analysis of genomic DNA was performed on the Illumina MiSeq platform (150 bp paired-end reads) (Illumina, Inc., San Diego, CA, USA) using the TruSight Cardio Sequencing Kit (Illumina, Inc., San Diego, CA, USA) for genetic profiling of 174 genes with known associations to 17 different inherited cardiac conditions (ICCs) [17].

Genomic DNA was isolated from the peripheral blood with the QIAamp DNA mini-kit (QIAGEN GmbH, Hilden, Germany) after obtaining informed consent. 

Data analysis was performed using the on-instrument MiSeq Reporter software 2.5.42.5 according to the BWA Enrichment workflow. Variant Studio (Illumina, Inc., San Diego, CA, USA) software and open-access bioinformatic tools and databases were used for the analysis and interpretation of variants obtained in the VCF file.

The interpretation of genetic variants was made according to ACMG/AMP guidelines [16,18].

All experimental procedures were performed according to guidelines and regulations and abided by the tenets of the Declaration of Helsinki.

## 3. Results

**Figure 1:**
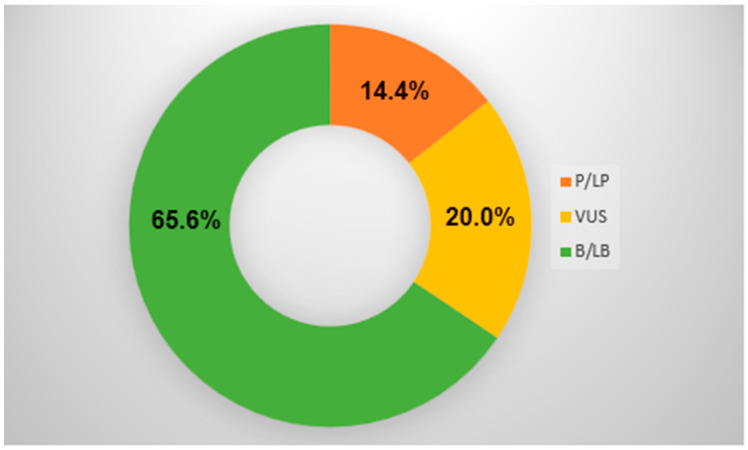
NGS outcome. P/LP = pathogenic/likely pathogenic variant; VUS = variant of unknown significance; B/LB = benign/likely benign variant.

From the cohort of 208 patients (Figure 1), genetic variants were detected in approximately 34.4% (72/208) of the study population, with a significant proportion of likely pathogenic or pathogenic variants (LP or P) of 14.4% (31/208) (Table 1) and 20% (42/208) of variants of unknown significance (VUS) (Table 2). In Table 1, seven variants were reclassified from LP to VUS variant and one variant from VUS to LP variant due to newly obtained data from the literature. In the pathogenic variant group and in the VUS group, 80% of patients were stratified for aggressive treatment of sudden cardiac death with ICD implantation as secondary prevention. Additionally, we selected four interesting patients with regard to their genetic test and clinical outcome: cases 1 and 2 with LB variants, case 3 where LP status was changed to VUS, and case 4 where VUS was changed to LP variant. 

**Case 1:** A female patient at the age of 20 years with a benign NM_033118.4:c.4G>A variant in the MYLK2 gene presented clinically as an arrhythmogenic variant of non-dilated left ventricular cardiomyopathy (NDVLC), treated clinically after aborted sudden death due to VF resuscitated to sinus rhythm from VF with complete neurological restitution. The patient was without clinical structural heart disease with normal LVEF, normal cardiac MRI targeted for fibrosis, and normal coronary angiography despite a normal 12-lead ECG ajmaline test performed with a normal outcome; on EPS and programmed stimulation, no ventricular arrhythmias were induced, and the test was declared as normal. NGS analysis showed the presence of the MYLK2(NM_033118.4):c.4G>A variant, first classified as VUS and later reclassified as a likely benign variant [19]. Regardless of the benign variant of the MYLK2 missense mutation due to clinical criteria in this patient as aborted sudden death, an ICD was implanted. After two years, two new VF episodes were set up during the night on resting conditions, both converted by ICD discharge into sinus rhythm. Both episodes set up during sinus rhythm without previously increased ventricular ectopic activity or non-sustained VT. After the second episode, an EPS was performed and was non-inducible, although an increased dispersion in refractoriness was present. The patient was clinically well under constant follow-ups every 6 months in an external clinic; no new episodes of VT or VF were present up to now, and no deterioration in LV function is present.

**Case 2:** A male patient at the age of 63 from the benign group is presented because of a survival of aborted sudden death with suspected arrhythmogenic cardiomyopathy, without present clinical structural heart disease, with normal echocardiography, a normal heart MRI, a normal CA, a normal ECG, and a normal non-inducible EPS test. NGS analysis showed the presence of TTN(NM_133378.4): c.7816G>A variant was first classified as VUS and later reclassified as a benign variant. A prophylactic ICD implantation was performed due to clinical risk stratification. Despite this, the patient developed overt dilative cardiomyopathy two years after ICD implantation, with a low LVEF < 25% and LBBB on ECG. Therefore, ICD was upgraded from a prophylactic single chamber device to cardiac resynchronization therapy (CRT-D), and the patient was scheduled for Tx therapy in the future. Presently, the patient is followed up at an external clinic and is on optimal medical therapy, as determined by CRT-D and carefully screened LV function.

**Case 3:** For a female patient at age 38 years (case No. 13 from Table 1), the LP variant MYBPC3(NM_000256.3):c.1219G>A was reclassified to the VUS variant [20,21]. In this case, the patient was admitted after severe syncope with borderline LV systolic function (EVEF 55%), normal CA, and non-inducible EPS and was treated with optimal medical therapy during the following two years. EVEF decreased to 30%, and left ventricular desynchronization on echocardiography with LBBB appeared. The patient was also reapproved by cardiac MRI; normal CA and episodes of monomorphic, non-sustained VT were present on EPS and were reproducible. Due to clinical criteria, a CRT-D device was implanted, and optimal medical therapy was continued. LVEF has improved from 30% to 50%, but the patient was four times cardioverted from sustained VT/VF by device. The patient is presently treated with optimal medical therapy, and radiofrequency ablation of arrhythmia has been performed due to several non-sustained VTs with partial success. Presently, the patient is stable on optimal medical therapy and on the antiarrhythmic medication amiodarone 200 mg/d without VT or VF and is scheduled for Tx in the future. Although the LP variant was, after a 3-year evaluation, classified as VUS according to ACMG criteria, it may play an important role in the clinical phenotype.

**Case 4:** A female patient at the age of 43 years (case No. 17 from Table 1) was admitted to the department after several severe syncopes. A typical ECG for LQTS type 2 was present. Several episodes of polymorphic self-terminating VT morphology in Torsade de points were observed. Before the described episodes, she was on a strong reduction diet due to obesity and a low serum potassium level, and the recurrence of episodes of torsade de pointes ceased with potassium level normalization. Despite the termination of polymorphic VT on Holter, an ECG typical for this syndrome was present. No structural disease, normal echocardiography, or normal CA were present. The patient was treated with standard medical therapy with β-blockers (Nadolol 80 mg daily), potassium supplements, and later a prophylactic double chamber ICD was implanted. Due to sinus bradycardia precipitated by β blockade with preserved AV conduction, the patient is paced in the AAIR (atrial pacing) modus by DR-ICD. No further episodes of polymorphic VT were present. NGS analysis showed the presence of the KCNH2(NM_000238.3):c.1863 C>G variant, the first classified as VUS, although typical ECG for LQTS 2 was present. After 3 years of re-evaluation, this variant was reclassified from VUS to pathogenic variant since it was reported as a genetic cause of Long QT syndrome in several patients [22,23,24,25]. Presently, the patient is well on pacing therapy by a double chamber ICD and by use of β-blocking medication.

The text continues here.

## 4. Discussion

Early cardiomyopathy detection is of great clinical significance for the prevention of further arrhythmias and SCD, the development of further structural changes, and also for the protection of relatives [26]. Searching for the underlying cause of VT or VF or even suspected clinically not developed cardiomyopathy due to unknown underlying causes has always been challenging, and in some cases, strongly suspected for probably rare Mendelian diseases [27,28,29]. Until today, in our clinical environment, molecular genetic diagnostic testing, especially in SCD survivors due to VT/VF, was rather limited due to complex and costly molecular genetic tests [30,31,32]. The most important genetic information was undiscovered, inadequate, or lost to use for counseling other family members. 

With the use of NGS analysis, personalized medicine has made tremendous progress, enabling a leap from the analysis of individual genes to the analysis of gene panels, with which we discover variants, from benign variants of unknown significance to pathogenic variants, that play a role in the development of cardiomyopathies. The variant interpretation is based on ACMG/AMP guidelines on clinical genetics and phenotype information, population data, computational predictive data, and characteristics of gene mutation [33].

The identification of pathogenic variants is not only important for patients but also enables the detection of risk in relatives, which is an additional challenge, and in many cases, an ICD implantation as antiarrhythmic medication is needed to prevent SCD even in those with mild clinical signs [34,35]. Several cases in our study with clear structural and/or electrophysiologic pathogenicity were declared as VUS and were carefully followed in the next few years [36].

We have selected four cases that we find interesting to describe regarding the difficulties in clinical decision-making and risk stratification after the NGS results. In all of them, the analyzed variants were reclassified over time. In Case 1, NGS analysis showed the presence of MYLK2(NM_033118.4):c.4G>A variant was first classified as VUS and later changed to a benign variant, although the patient had a very malignant clinical course. Although cardiac myosin light chain kinase has been implicated in cardiac adaptation to oxidative stress in some in vitro and animal studies, no clinical data have been reported for this gene mutation [28,37,38]. According to the ESC Guidelines (ref.), the MYLK2 gene is not associated with NDVLC. In our patient, it is possible that some other gene, which was not included in our gene panel, was playing a role. A WES analysis should be performed on this patient. In Case 2, NGS analysis showed the presence of TTN(NM_133378.4): c.7816G>A variant, which was classified first as VUS and later changed to a benign variant. Despite the genetic classification, the patient experienced an aborted, sudden death. The TTN gene is commonly involved in DCM, with strong evidence according to ASC Guidelines [2], but not the variant found in our patient. Secondary risk factors influence the penetrance and expressivity of TTNtvs [39]. In our patient, it is possible that his lifestyle affected the malignant course of the TTN variant. In Case 3, NGS analysis in a highly symptomatic patient showed the presence of MYBPC3(NM_000256.3): c.1219G>A variant was first classified as LP and later changed to VUS. In this case, the clinical diagnosis was obvious: LV systolic dysfunction, low LVEF and LBBB, and episodes of monomorphic, non-sustained VT [40,41]. The patient responded to optimal medical therapy and implanted CRT-D, which is scheduled for Tx. In Case 4, the patient was treated first by standard medical therapy with β-blockers (Nadolol 80 mg daily), eplerenone 25 mg daily, and potassium supplements. NGS analysis showed the presence of the KCNH2(NM_000238.3):c.1863 C>G VUS variant, which was after 3 years reclassified from VUS to a P variant. In the following years, a prophylactic double chamber device (DR-ICD) was implanted [35]. 

Although prophylactic ICD implantation or aggressive electrophysiological testing after aborted sudden death is established under clinical criteria and guidelines [11,20], the majority of the patients are asymptomatic in the following years and are followed under the rather nonspecific diagnosis of “idiopathic VF”. When a pathogenic variant, or, as in several of our cases, VUS, is determined by NGS [12,42], a patient is followed up as having a determined disorder, and a more precise and specific diagnosis is established, which could serve to prevent and provide more specific treatment for the patient and their relatives.

In the study, we established a molecular genetic disorder in 34.4% of the study population. A significant proportion of 14.4% of pathogenic and 20% of VUS variants was analyzed. In a group of patients with VUS, 80% were highly clinically significant, and the correlation between genotype and phenotype was established and discussed [30]. The emphasis of the study is placed on the importance of the results of NGS analyses in defining the clinical condition and risk stratification, with the awareness that early cardiomyopathy detection, aggressive treatment, and SCD prevention are of great clinical significance [26,27].

The problem remains with VUS and benign variants [33,42]. We have observed that some VUS variants (3.4%) in our group of patients change their status in the following years to pathogenic, and there was one case where a pathogenic variant shifted to VUS. On the other hand, in the VUS group, 32% of patients protected by ICD under clinical criteria remain highly symptomatic, with multiple episodes of ventricular tachyarrhythmias recorded and treated by an implantable ICD device. Therefore, some VUS cases should be carefully observed and clinically followed up; the classification of variants may change based on new data from described cases, the identification of causative genes, or new functional studies [12,29]. Reclassifications from VUS to pathogenic variants and from VUS to benign variants are common [42], but rarely from benign to VUS or pathogenic variants. The problem remains with those benign variants that are not subject to reanalysis in the following years. According to the clinical presentation of the patients, the requalification of benign variants into VUS or even pathogenic ones could certainly occur in certain cases. Certainly, with an expanded set of genes using whole exome/genome WES/WGS testing, other genetic causes of the disease could be found in many clinical cases. The correct classification of variants is necessary to guide personalized decisions in the treatment of patients and the management of relatives and asymptomatic people. Therefore, regular reanalysis and reinterpretation of the variants is necessary. The problem remains mainly VUS variants, which are not clinically actionable [15].

## 5. Conclusions

The shortcoming of the presented study is that, at the time of the study, we had used a gene panel of a limited number of genes associated with cardiomyopathies, and we did not have WES/WGS at our disposal. Technologies such as WES/WGS would also enable us to detect smaller deletions or duplications that can affect the proper functioning of disease-associated genes. Even in patients with VUS variants, testing with WES/WGS could lead to the detection of other variants that were missed due to the limitations of our gene panel. In addition, in patients with benign variants and patients without identified variants, testing a wider range of cardiac disease-related genes (such as FLNC, ALPK3, CDH, etc.) not included in our study would lead to greater utility of molecular analysis.

## Figures and Tables

**Table 1:** Representative data for clinical validation—likely pathogenic and pathogenic variants.

Patient	Gene	Variant (Assembly UCSC, hg38)	Transcript Change	Protein Change	Variant Type
1	MYH7	chr14:23429005 G-A	NM_000257.4:c.1357C>T	p.(Arg453Cys)	missense
2 *	LMNA	chr1:g. 156136287 G-T	NM_170707.4:c.1231G>T	p.(Gly411Cys)	missense
3	MYBPC3	chr11:g. 47335082 AG-	NM_000256.3:c.2864_2865delCT	p.(Pro955ArgfsTer95)	fremeshift
4 *	LMNA	chr1:g. 156136287 G-T	NM_170707.4:c.1231G>T	p.(Gly411Cys)	missense
5 *	JPH2	chr20:g. 44115694 C-	NM_020433.4:c.1981delG	p.(Ala661ArgfsTer20)	fremeshift
6	MYBPC3	chr11:g. 47337467 G-C	NM_000256.3:c.2526C>G	p.(Tyr842Ter)	sStop gain
7	SCN5A	chr3:g. 38606034 G-A	NM_198056.2:c.1255C>T	p.(Gln419Ter)	stop gain
8	KCNH2	chr7:g. 150951679 C-T	NM_000238.3:c.1714G>A	p.(Gly572Ser)	missense
9	MYBPC3	chr11:g. 47342718 C-T	NM_000256.3:c.1484G>A	p.(Arg495Gln)	missense
10	MYH6	chr14:g. 23405122 C-A	NM_002471.3:c.508G>T	p.(Glu170Ter)	stop gain
11	TTN	chr2:g. 178575999 C-T	NM_001267550.1:c.70133G>A	p.(Trp23378Ter)	stop gain
12	VCL	chr10:g. 74071036 --T	NM_014000.3:c.452dup	p.(Glu152GlyfsTer19)	insertion
13 *	MYBPC3	chr11:g. 47343496 C-T	NM_000256.3:c.1219G>A	p.(Gly407Ser)	missense
14	LMNA	chr1:g. 156134819 C-	NM_170707.3:c.654delC	p.(Lys219SerfsTer261)	deletion
15	MYH7	chr14:g. 23428516 A-G	NM_000257.2:c.1562T>C	p.(Ile521Thr)	missense
16	PKP2	chr12:g. 32796145 C-	NM_004572.3:c.2453delG	p.(Gly818AlafsTer113)	deletion
17 **	KCNH2	chr7:g. 150951530 G-C	NM_000238.4:c.1863C>G	p.(Ser621Arg)	missense
18	TTN	chr2:g. 178571794 G-A	NM_001256850.1:c.69415C>T	p.(Arg23139Ter)	stop gain
19	LMNA	chr1:g. 156134819 C-	NM_170707.3:c.654delC	p.(Lys219SerfsTer261)	deletion
20	DSP	chr6:g. 7555821 G-A	NM_004415.2:c.273+1G>A	p.?	alternative splicing
21	MYBPC3	chr11:g. 47337467 G-C	NM_000256.3:c.2526C>G	p.(Tyr842Ter)	stop gain
22	GJA5	chr1:g 147758631 T-G	NM_005266.6:c.608A>C	p.(Glu203Ala)	missense
23	LMNA	chr1:g 156134518 T-C	NM_170707.4:c.629T>C	p.(Ile210Thr)	missense
24 *	RAF1	chr3:g. 12585165 A-G	NM_002880.4:c.1625T>C	p.(Met542Thr)	missense
25	TTN	chr2:g. 178799908 C-A	NM_001267550.2:c.586G>T	p.(Glu196Ter)	stop gain
26	BRAF	chr7:g. 140801542 T-C	NM_004333.6:c.730A>G	p.(Thr244Ala)	missense
27	TTN	chr2:g. 178542496 G-	NM_001267550.2:c.97260del	p.(Trp32421GlyfsTer12)	fremeshift
28 *	TTN	chr2:g. 178584435 A-G	NM_001267550.1:c.65116T>C	p.(Trp21706Arg)	missense
29	MYH7	chr14:g. 23429262 A-T	NM_000257.2:c.1224T>A	p.(Asn408Lys)	missense
30 *	MYBPC3	chr11:g. 47335970 A-G	NM_000256.3:c.2644T>C	p.(Ser882Pro)	missense

* Cases in which, after 2–3 years, the classification of the variant changed from LIKELY PATHOGEN to VUS. ** Case in which, after 2–3 years, the classification of the variant changed from VUS to LIKELY PATHOGEN.

**Table 2:** Representative data for clinical validation—VUS variants.

Patient	Gene	Variant (Assembly UCSC, hg38)	Transcript Change	Protein Change	Variant Type
1	TTN	chr2:g. 178567143 C-T	NM_001267550.1:c.78989G>A	p.(Ser26330Asn)	missense
2	TTN	chr2:g. 178572357 C-G	NM_001267550.1:c.73775G>C	p.(Arg24592Thr)	missense
3	TTN	chr2:g. 178720616 C-A	NM_001267550.1:c.23146G>T	p.(Gly7716Cys)	missense
4	MYH6	chr14:g. 23396970 G-A	NM_002471.3:c.2161C>T	p.(Arg721Trp)	missense
5	MYH6	chr14:g. 23397017 C-T	NM_002471.3:c.2114G>A	p.(Arg705His)	missense
6	PRKAG2	chr7:g. 151565804 T-C	NM_016203.3:c.1315A>G	p.(Ile439Val)	missense
7	KCNQ1	chr11:g. 2588836 G-A	NM_000218.2:c.1375G>A	p.(Asp459Asn)	missense
8	LDB3	chr10:g. 86718073 G-A	NM_001171610.1:c.1801G>A	p.(Val596Ile)	missense
9 *	ACTA1	chr1:g. 229431782 C-T	NM_001100.3:c.929G>A	p.(Gly310Glu)	missense
10	LDB3	chr10:g. 86718073 G-A	NM_001171610.1:c.1801G>A	p.(Val601Ile)	missense
11	JUP	chr17:g. 41769629 C-T	NM_002230.4:c.257G>A	p.(Arg86Gln)	missense
12	TTN	chr2:g. 178567143 C-T	NM_001267550.1:c.78989G>A	p.(Ser26330Asn)	missense
13	KCND3	chr1:g. 11177845 G-A	NM_004980.5:c.1501C>T	p.(Arg501Ter)	stop-gain
14	CASQ2 TTN	chr1:g. 115732965G-C chr2:g. 178545597 T-C	NM_001232.3:c.542C>G NM_001267550.1:c.95513A>G	p.(Ala181Gly) p.(Glu31838Gly)	missense missense
15	RYR2	chr1:g. 237698992 G-C	NM_001035.3:c.9095G>C	p.(Cys3032Ser)	missense
16	KCNE3	chr11:g. 74457269 G-A	NM_005472.4:c.295C>T	p.(Arg99Cys)	missense
17	RAF1	chr3:g. 12611956 T-A	NM_002880.3:c.314A>T	p.(His105Leu)	missense
18	CRP3	chr11:g. 19185024 G-A	NM_003476.5:c.436C>T	p.(Arg146Cys)	missense
19	TNNT2 MYH7	chr1:g. 201363322T-C chr14:g. 23423938 A-G	NM_001276345.2:c.574A>G NM_000257.4:c.2891T>C	p.(Met192Val) p.(Val964Ala)	missense missense
20	MYH6	chr14:g. 23389018 C-A	NM_002471.4:c.4016G>T	p.(Arg1339Leu)	missense
21	MYH11	chr16:g. 15717232 C-T	NM_001040114.1:c.5433G>A	p.(Met1811Ile)	missense
22	DSP	chr6:g. 7579655 G-C	NM_004415.4:c.3465G>C	p.(Trp1155Cys)	missense
23	DSG2	chr18:g. 31546084 G-C	NM_001943.5:c.2698G>C	p.(Glu900Gln)	missense
24	NEXN1	chr1:g. 77942606 C-T	NM_144573.4:c.1805C>T	p.(Thr602Met)	missense
25	RYR2	chr1:g. 237674110 C-T	NM_001035.3:c.8605C>T	p.(Pro2869Ser)	missense
26	CACNA1C	chr12:g. 2566445 G-A	NM_199460.3:c.1532G>A	p.(Arg511Gln)	missense
27	MYH6	chr14:g. 23407028 C-T	NM_002471.4:c.196G>A	p.(Gly66Arg)	missense
28	TTN	chr2:g. 178718874 A-C	NM_001256850.1:c.23375T>G	p.(Val7792Gly)	missense
29	PKP2	chr12:g. 32841047 T-C	NM_004572.4:c.1669A>G	p.(Asn557Asp)	missense
30	MYH11	chr16:g. 15745209 T-C	NM_022844.2:c.2440A>G	p.(Thr814Ala)	missense
31	CACNA1C	chr12:g. 2053575 A-C	NM_199460.3:c.13A>C	p.(Asn5His)	missense
32	NOTCH1	chr9:g. 136510685 T-C	NM_017617.5:c.2708A>G	p.(Asn903Ser)	missense
33	DSC2	chr18:g.31074895 C-T	NM_024422.6:c.1676G>A	p.(Cys559Tyr)	missense
34	TNNI3	chr19:g. 55157079 G-T	NM_000363.5:c.79C>A	p.(Arg27Ser)	missense
35 **	JUP	chr17:g. 41769150 G-A	NM_002230.4:c.526C>T	p.(Arg176Trp)	missense
36	MYH11	chr16:g. 15784699 T-C	NM_001040114.1:c.653A>G	p.(Tyr218Cys)	missense
37	LMNA	chr1:g. 156135992 G-A	NM_170707.4:c.1028G>A	p.(Arg343Gln)	missense
38	LDB3	chr10:g. 86692555 G-A	NM_001171610.2:c.1084G>A	p.(Ala362Thr)	missense
39 **	ACTC1	chr15:g. 34792247 C-A	NM_005159.5:c.651C>T	p.(Lys217Asn)	missense
40	COL5A1	chr9:g. 134700094 T-C	NM_001278074.1:c.463T>C	p.(Phe155Leu)	missense
41	DSP	chr6:g. 7585657 G-A	NM_004415.4:c.8395G>A	p.(Gly2799Arg)	missense
42	MYBPC3	chr11:g. 47335970 A-G	NM_000256.3:c.2644T>C	p.(Ser882Pro)	missense

* Case in which, after 2–3 years, the classification of the variant changed from VUS to LIKELY PATHOGENIC. ** Cases in which, after 2–3 years, the classification of the variant changed from VUS to LIKELY BENIGN.

## Data Availability

All the relevant data have been provided in the manuscript, and any supplementary datasets used and/or analyzed during the current study are available from the corresponding author upon reasonable request.
